# Supplementary material for: Nausea and Vomiting during Early Pregnancy among Chinese Women and Its Association with Nutritional Intakes
Source: Nutrients. 2023 Feb 13;15(4):933. doi: 10.3390/nu15040933 (PMC9962185; doi:10.3390/nu15040933)
Supplement: Supplementary file 1 [file nutrients-15-00933-s001.zip › nutrients-2088494-supplementary.pdf]

Table S1 The food group classification standard

Cereals	Rice and its products (rice, rice flour, etc.); Wheat and its products (steamed bread, noodles, etc.); Miscellaneous grains and other cereals (corn, millet, buckwheat, etc.)
Potatoes	Sweet potato, potato, taro, yam, etc.
Fresh vegetables	Leaf vegetables, rhizomes, melons and eggplants, etc
Pickled and fermented vegetables	Such as pickled cucumber and fermented Chinese cabbage
Mushroom and algae	Mushrooms (Lentinus edodes, Hericium erinaceus, etc.); Agaric, tremella, etc; Kelp; Laver;
Soybeans and their products	Soybean, bean curd, bean curd, soybean milk, etc
Nuts and seeds	Walnuts, melon seeds, peanuts, etc
Fruits	Apple, orange, strawberry, papaya, banana, etc
Poultry meat	Pork, beef, mutton, donkey meat, etc
Livestock meat	Chicken, duck, goose, etc
Aquatic products	Fish, shrimp, shellfish, crab, etc
Eggs	Eggs, duck eggs, preserved eggs, etc
Dairy products	Pure milk, yoghurt, milk powder, other dairy products
Soups	Broth, bone soup, fish soup, etc
Snacks and beverages	Beverages, tea, coffee, etc;<br>Cake (cake, biscuit, Chinese dessert, etc.); Candy candies; Chocolates; Puffed food; Other snacks

Table S2 Results of multiple regression model with daily nutrient intake as the dependent variable

(a) Energy(kcal)

Variables		β	95% CI		Wald value	P
			Lower Limit	Upper Limit		
NVP group						
	NVP	-299.271	-510.817	-87.725	7.688	0.006
	Non-NVP	Ref.				
Age (years)						
	< 30	6.041	-170.874	182.955	.004	0.947
	≥30	Ref.				
Education						
	Junior high school or below	-190.786	-431.161	49.589	2.420	0.120
	High school / technical secondary school	-36.673	-234.606	161.259	.132	0.716
	College degree or above	Ref.				
Family monthly per capita income						
	< 5000	-154.885	-386.666	76.897	1.715	0.190
	5000–9999	-194.578	-411.936	22.781	3.078	0.079
	≥10000	Ref.				
Cities						
	First-tier	-151.112	-392.010	89.786	1.512	0.219
	New first-tier	-308.594	-537.444	-79.743	6.985	0.008
	Second-tier	-40.524	-263.342	182.294	.127	0.721
	Third- or fourth-tier	Ref.				
Pre-pregnancy BMI						
	Underweight	186.418	-126.009	498.845	1.368	0.242
	Normal	5.285	-191.442	202.012	.003	0.958
	Overweight/obesity	Ref.				
Gravidity						
	1	-68.986	-283.932	145.960	.396	0.529
	≥2	Ref.				
Parity						
	0	-36.224	-270.357	197.908	.092	0.762
	≥1	Ref.				

(b) Protein(g)

Variables		β	95% CI		Wald value	P
			Lower Limit	Upper Limit		
NVP group						
	NVP	-18.57	-29.084	-8.056	11.985	0.001
	Non-NVP	Ref.				
Age (years)						
	< 30	3.903	-4.89	12.695	0.757	0.384
	≥30	Ref.				

Education					
Junior high school or below	-15.428	-27.375	-3.482	6.407	0.011
High school / technical secondary school	-2.991	-12.828	6.846	0.355	0.551
College degree or above	Ref.				
Family monthly per capita income					
< 5000	-5.351	-16.87	6.168	0.829	0.363
5000–9999	-9.96	-20.762	0.843	3.266	0.071
≥10000	Ref.				
Cities					
First-tier	1.339	-10.634	13.311	0.048	0.827
New first-tier	-4.079	-15.453	7.295	0.494	0.482
Second-tier	2.77	-8.304	13.843	0.24	0.624
Third- or fourth-tier	Ref.				
Pre-pregnancy BMI					
Underweight	14.549	-0.978	30.076	3.373	0.066
Normal	1.619	-8.158	11.396	0.105	0.745
Overweight/obesity	Ref.				
Gravidity					
1	1.012	-9.671	11.694	0.034	0.853
≥2	Ref.				
Parity					
0	-8.936	-20.572	2.7	2.266	0.132
≥1	Ref.				

(c) Fat(g)

Variables	β	95% CI		Wald value	P
		Lower Limit	Upper Limit		
NVP group					
NVP	-16.419	-25.712	-7.127	11.994	0.001
Non-NVP	Ref.				
Age (years)					
< 30	2.617	-5.154	10.388	0.436	0.509
≥30	Ref.				
Education					
Junior high school or below	-10.524	-21.082	0.035	3.816	0.051
High school / technical secondary school	-6.665	-15.360	2.029	2.258	0.133
College degree or above	Ref.				
Family monthly per capita income					
< 5000	-6.556	-16.737	3.625	1.593	0.207
5000–9999	-5.370	-14.918	4.177	1.215	0.270
≥10000	Ref.				
Cities					
First-tier	-14.101	-24.683	-3.520	6.822	0.009

New first-tier	-3.591	-13.644	6.461	0.490	0.484
Second-tier	-8.332	-18.120	1.455	2.784	0.095
Third- or fourth-tier	Ref.				
Pre-pregnancy BMI					
Underweight	10.064	-3.660	23.787	2.066	0.151
Normal	1.958	-6.683	10.599	0.197	0.657
Overweight/obesity	Ref.				
Gravidity					
1	-3.028	-12.470	6.413	0.395	0.530
≥2	Ref.				
Parity					
0	-3.703	-13.987	6.582	0.498	0.480
≥1	Ref.				

(d) Carbohydrate(g)

Variables	β	95% CI		Wald value	P
		Lower Limit	Upper Limit		
NVP group					
NVP	-25.641	-57.720	6.438	2.454	0.117
Non-NVP	Ref.				
Age (years)					
< 30	-1.247	-28.075	25.580	0.008	0.927
≥30	Ref.				
Education					
Junior high school or below	-4.291	-40.742	32.159	0.053	0.818
High school / technical secondary school	10.946	-19.069	40.960	0.511	0.475
College degree or above	Ref.				
Family monthly per capita income					
< 5000	-5.599	-40.747	29.549	0.097	0.755
5000–9999	-20.172	-53.133	12.788	1.439	0.230
≥10000	Ref.				
Cities					
First-tier	0.337	-36.193	36.867	0.000	0.986
New first-tier	-49.674	-84.377	-14.971	7.871	0.005
Second-tier	15.830	-17.958	49.619	0.843	0.358
Third- or fourth-tier	Ref.				
Pre-pregnancy BMI					
Underweight	22.582	-24.794	69.959	0.873	0.350
Normal	3.560	-26.272	33.392	0.055	0.815
Overweight/obesity	Ref.				
Gravidity					
1	4.391	-28.203	36.986	0.070	0.792
≥2	Ref.				

Parity					
0	-5.513	-41.017	29.992	0.093	0.761
≥1	Ref.				

(e) Dietary fiber(g)

Variables	β	95% CI		Wald value	P
		Lower Limit	Upper Limit		
NVP group					
NVP	-2.561	-5.248	0.126	3.489	0.062
Non-NVP	Ref.				
Age (years)					
< 30	-1.999	-4.246	0.248	3.039	0.081
≥30	Ref.				
Education					
Junior high school or below	-2.253	-5.307	0.800	2.092	0.148
High school / technical secondary school	0.297	-2.217	2.811	0.054	0.817
College degree or above	Ref.				
Family monthly per capita income					
< 5000	2.176	-0.768	5.120	2.098	0.147
5000–9999	0.476	-2.285	3.237	0.114	0.735
≥10000	Ref.				
Cities					
First-tier	0.753	-2.307	3.813	0.232	0.630
New first-tier	1.573	-1.334	4.480	1.124	0.289
Second-tier	1.676	-1.154	4.507	1.347	0.246
Third- or fourth-tier	Ref.				
Pre-pregnancy BMI					
Underweight	4.303	0.334	8.271	4.516	0.034
Normal	1.149	-1.350	3.648	0.812	0.368
Overweight/obesity	Ref.				
Gravidity					
1	0.466	-2.265	3.196	0.112	0.738
≥2	Ref.				
Parity					
0	-0.067	-3.041	2.907	0.002	0.965
≥1	Ref.				

(f) Cholesterol(mg)

Variables	β	95% CI		Wald value	P
		Lower Limit	Upper Limit		
NVP group					
NVP	-87.567	-197.351	22.216	2.444	0.118
Non-NVP	Ref.				

Age (years)					
< 30	86.634	-5.177	178.446	3.420	0.064
≥30	Ref.				
Education					
Junior high school or below	-136.780	-261.525	-12.035	4.618	0.032
High school / technical secondary school	-138.208	-240.927	-35.490	6.954	0.008
College degree or above	Ref.				
Family monthly per capita income					
< 5000	10.220	-110.066	130.505	0.028	0.868
5000–9999	20.637	-92.163	133.437	0.129	0.720
≥10000	Ref.				
Cities					
First-tier	134.804	9.787	259.820	4.466	0.035
New first-tier	77.616	-41.148	196.380	1.641	0.200
Second-tier	54.981	-60.653	170.614	0.868	0.351
Third- or fourth-tier	Ref.				
Pre-pregnancy BMI					
Underweight	0.204	-161.933	162.341	0.000	0.998
Normal	32.570	-69.524	134.663	0.391	0.532
Overweight/obesity	Ref.				
Gravidity					
1	121.407	9.859	232.955	4.550	0.033
≥2	Ref.				
Parity					
0	-181.074	-302.580	-59.569	8.531	0.003
≥1	Ref.				

(g) Vitamin A(μgREA)

Variables	β	95% CI		Wald value	P
		Lower Limit	Upper Limit		
NVP group					
NVP	-95.335	-355.980	165.310	0.514	0.473
Non-NVP	Ref.				
Age (years)					
< 30	-127.423	-345.399	90.552	1.313	0.252
≥30	Ref.				
Education					
Junior high school or below	122.025	-174.140	418.189	0.652	0.419
High school / technical secondary school	-27.907	-271.778	215.965	0.050	0.823
College degree or above	Ref.				
Family monthly per capita income					
< 5000	15.651	-269.926	301.229	0.012	0.914
5000–9999	-133.581	-401.387	134.226	0.956	0.328

≥10000	Ref.				
Cities					
First-tier	42.130	-254.679	338.940	0.077	0.781
New first-tier	-27.571	-309.536	254.394	0.037	0.848
Second-tier	214.505	-60.028	489.038	2.345	0.126
Third- or fourth-tier	Ref.				
Pre-pregnancy BMI					
Underweight	-287.773	-672.712	97.167	2.147	0.143
Normal	-261.506	-503.892	-19.120	4.471	0.034
Overweight/obesity	Ref.				
Gravidity					
1	93.244	-171.591	358.078	0.476	0.490
≥2	Ref.				
Parity					
0	-163.617	-452.091	124.856	1.236	0.266
≥1	Ref.				

(h) Thiamin(mg)

Variables	β	95% CI		Wald value	P
		Lower Limit	Upper Limit		
NVP group					
NVP	-0.210	-0.359	-0.060	7.577	0.006
Non-NVP	Ref.				
Age (years)					
< 30	0.051	-0.073	0.176	0.651	0.420
≥30	Ref.				
Education					
Junior high school or below	-0.234	-0.404	-0.064	7.291	0.007
High school / technical secondary school	-0.073	-0.213	0.066	1.060	0.303
College degree or above	Ref.				
Family monthly per capita income					
< 5000	0.004	-0.160	0.168	0.002	0.962
5000–9999	-0.067	-0.220	0.087	0.723	0.395
≥10000	Ref.				
Cities					
First-tier	0.080	-0.090	0.250	0.848	0.357
New first-tier	0.025	-0.137	0.187	0.092	0.762
Second-tier	0.085	-0.072	0.242	1.124	0.289
Third- or fourth-tier	Ref.				
Pre-pregnancy BMI					
Underweight	0.055	-0.165	0.276	0.241	0.623
Normal	-0.027	-0.166	0.112	0.146	0.703
Overweight/obesity	Ref.				

Gravidity					
1	0.059	-0.093	0.211	0.581	0.446
≥2	Ref.				
Parity					
0	-0.177	-0.342	-0.011	4.383	0.036
≥1	Ref.				

(i) Riboflavin(mg)

Variables	β	95% CI		Wald value	P
		Lower Limit	Upper Limit		
NVP group					
NVP	-0.346	-0.531	-0.160	13.355	< 0.001
Non-NVP	Ref.				
Age (years)					
< 30	0.050	-0.105	0.205	0.401	0.527
≥30	Ref.				
Education					
Junior high school or below	-0.155	-0.366	0.056	2.081	0.149
High school / technical secondary school	-0.010	-0.184	0.163	0.014	0.906
College degree or above	Ref.				
Family monthly per capita income					
< 5000	0.014	-0.189	0.217	0.019	0.890
5000–9999	-0.001	-0.192	0.189	0.000	0.990
≥10000	Ref.				
Cities					
First-tier	0.039	-0.172	0.250	0.130	0.718
New first-tier	-0.013	-0.214	0.187	0.017	0.896
Second-tier	0.103	-0.092	0.299	1.077	0.299
Third- or fourth-tier	Ref.				
Pre-pregnancy BMI					
Underweight	0.040	-0.234	0.313	0.080	0.777
Normal	-0.019	-0.191	0.153	0.047	0.828
Overweight/obesity	Ref.				
Gravidity					
1	0.153	-0.036	0.341	2.518	0.113
≥2	Ref.				
Parity					
0	-0.223	-0.429	-0.018	4.556	0.033
≥1	Ref.				

(j) Niacin(mg)

Variables	β	95% CI		Wald value	P
		Lower Limit	Upper Limit		

NVP group					
NVP	-1.918	-4.518	0.682	2.090	0.148
Non-NVP	Ref.				
Age (years)					
< 30	0.255	-1.920	2.429	0.053	0.818
≥30	Ref.				
Education					
Junior high school or below	-3.582	-6.536	-0.628	5.648	0.017
High school / technical secondary school	-1.771	-4.204	0.661	2.037	0.154
College degree or above	Ref.				
Family monthly per capita income					
< 5000	-2.880	-5.728	-0.031	3.926	0.048
5000–9999	-3.070	-5.741	-0.399	5.073	0.024
≥10000	Ref.				
Cities					
First-tier	1.495	-1.466	4.456	0.979	0.322
New first-tier	0.414	-2.399	3.227	0.083	0.773
Second-tier	1.809	-0.929	4.548	1.677	0.195
Third- or fourth-tier	Ref.				
Pre-pregnancy BMI					
Underweight	2.152	-1.688	5.992	1.207	0.272
Normal	0.508	-1.910	2.926	0.169	0.681
Overweight/obesity	Ref.				
Gravidity					
1	0.093	-2.549	2.735	0.005	0.945
≥2	Ref.				
Parity					
0	-3.226	-6.104	-0.348	4.828	0.028
≥1	Ref.				

(k) Vitamin C(mg)

Variables	β	95% CI		Wald value	P
		Lower Limit	Upper Limit		
NVP group					
NVP	4.603	-32.283	41.489	0.060	0.807
Non-NVP	Ref.				
Age (years)					
< 30	19.155	-11.692	50.003	1.481	0.224
≥30	Ref.				
Education					
Junior high school or below	-24.951	-66.863	16.962	1.361	0.243
High school / technical secondary school	12.578	-21.934	47.090	0.510	0.475
College degree or above	Ref.				

Family monthly per capita income					
< 5000	8.539	-31.875	48.953	0.171	0.679
5000–9999	18.418	-19.481	56.317	0.907	0.341
≥10000	Ref.				
Cities					
First-tier	11.292	-30.712	53.296	0.278	0.598
New first-tier	5.626	-34.277	45.529	0.076	0.782
Second-tier	-4.982	-43.833	33.870	0.063	0.802
Third- or fourth-tier	Ref.				
Pre-pregnancy BMI					
Underweight	6.583	-47.893	61.058	0.056	0.813
Normal	0.143	-34.159	34.445	0.000	0.993
Overweight/obesity	Ref.				
Gravidity					
1	35.021	-2.458	72.499	3.354	0.067
≥2	Ref.				
Parity					
0	4.603	-32.283	41.489	0.060	0.807
≥1	Ref.				

(l) Vitamin E(mg)

Variables	β	95% CI		Wald value	P
		Lower Limit	Upper Limit		
NVP group					
NVP	-7.211	-12.836	-1.586	6.312	0.012
Non-NVP	Ref.				
Age (years)					
< 30	-2.027	-6.731	2.677	0.713	0.398
≥30	Ref.				
Education					
Junior high school or below	-1.033	-7.425	5.358	0.100	0.751
High school / technical secondary school	1.252	-4.011	6.515	0.217	0.641
College degree or above	Ref.				
Family monthly per capita income					
< 5000	2.779	-3.384	8.942	0.781	0.377
5000–9999	1.990	-3.789	7.770	0.456	0.500
≥10000	Ref.				
Cities					
First-tier	-5.134	-11.539	1.272	2.468	0.116
New first-tier	0.599	-5.486	6.685	0.037	0.847
Second-tier	-1.321	-7.246	4.603	0.191	0.662
Third- or fourth-tier	Ref.				
Pre-pregnancy BMI					

Underweight	4.443	-3.865	12.750	1.099	0.295
Normal	1.288	-3.943	6.519	0.233	0.629
Overweight/obesity	Ref.				
Gravidity					
1	-2.526	-8.242	3.189	0.751	0.386
≥2	Ref.				
Parity					
0	1.801	-4.424	8.027	0.322	0.571
≥1	Ref.				

(m) Folate(μgDFE)

Variables	β	95% CI		Wald value	P
		Lower Limit	Upper Limit		
NVP group					
NVP	-36.848	-89.045	15.348	1.914	0.166
Non-NVP	Ref.				
Age (years)					
< 30	-2.445	-46.260	41.371	0.012	0.913
≥30	Ref.				
Education					
Junior high school or below	-77.769	-136.997	-18.542	6.623	0.010
High school / technical secondary school	-4.100	-53.565	45.364	0.026	0.871
College degree or above	Ref.				
Family monthly per capita income					
< 5000	47.612	-9.809	105.033	2.641	0.104
5000–9999	17.261	-36.771	71.293	0.392	0.531
≥10000	Ref.				
Cities					
First-tier	37.382	-22.307	97.070	1.507	0.220
New first-tier	15.162	-41.820	72.144	0.272	0.602
Second-tier	5.577	-49.691	60.844	0.039	0.843
Third- or fourth-tier	Ref.				
Pre-pregnancy BMI					
Underweight	70.644	-6.501	147.789	3.221	0.073
Normal	8.202	-40.540	56.943	0.109	0.742
Overweight/obesity	Ref.				
Gravidity					
1	30.457	-22.987	83.900	1.248	0.264
≥2	Ref.				
Parity					
0	-64.431	-122.606	-6.256	4.712	0.030
≥1	Ref.				

(n) Calcium(mg)

Variables	β	95% CI		Wald value	P
		Lower Limit	Upper Limit		
NVP group					
NVP	-200.839	-327.178	-74.500	9.708	0.002
Non-NVP	Ref.				
Age (years)					
< 30	45.076	-60.581	150.732	0.699	0.403
≥30	Ref.				
Education					
Junior high school or below	-117.611	-261.167	25.945	2.578	0.108
High school / technical secondary school	71.931	-46.278	190.140	1.422	0.233
College degree or above	Ref.				
Family monthly per capita income					
< 5000	-59.492	-197.917	78.932	0.710	0.400
5000–9999	-77.307	-207.117	52.504	1.362	0.243
≥10000	Ref.				
Cities					
First-tier	-37.045	-180.913	106.824	0.255	0.614
New first-tier	-101.006	-237.679	35.667	2.098	0.147
Second-tier	-24.039	-157.109	109.032	0.125	0.723
Third- or fourth-tier	Ref.				
Pre-pregnancy BMI					
Underweight	196.669	10.082	383.255	4.268	0.039
Normal	4.319	-113.170	121.808	0.005	0.943
Overweight/obesity	Ref.				
Gravidity					
1	48.721	-79.648	177.091	0.553	0.457
≥2	Ref.				
Parity					
0	-56.350	-196.178	83.478	0.624	0.430
≥1	Ref.				

(o) Phosphorus(mg)

Variables	β	95% CI		Wald value	P
		Lower Limit	Upper Limit		
NVP group					
NVP	-256.799	-392.773	-120.825	13.702	< 0.001
Non-NVP	Ref.				
Age (years)					
< 30	31.766	-81.948	145.480	0.300	0.584
≥30	Ref.				
Education					

Junior high school or below	-186.219	-340.723	-31.715	5.580	0.018
High school / technical secondary school	-36.801	-164.024	90.423	0.321	0.571
College degree or above	Ref.				
Family monthly per capita income					
< 5000	-62.539	-211.520	86.442	0.677	0.411
5000–9999	-100.610	-240.320	39.100	1.992	0.158
≥10000	Ref.				
Cities					
First-tier	22.299	-132.541	177.140	0.080	0.778
New first-tier	-90.727	-237.823	56.370	1.461	0.227
Second-tier	17.237	-125.982	160.457	0.056	0.814
Third- or fourth-tier	Ref.				
Pre-pregnancy BMI					
Underweight	190.025	-10.791	390.841	3.440	0.064
Normal	29.192	-97.257	155.641	0.205	0.651
Overweight/obesity	Ref.				
Gravidity					
1	60.434	-77.725	198.594	0.735	0.391
≥2	Ref.				
Parity					
0	-140.245	-290.737	10.247	3.336	0.068
≥1	Ref.				

(p) Potassium(mg)

Variables	β	95% CI		Wald value	P
		Lower Limit	Upper Limit		
NVP group					
NVP	-462.701	-843.736	-81.665	5.665	0.017
Non-NVP	Ref.				
Age (years)					
< 30	-28.026	-346.684	290.631	0.030	0.863
≥30	Ref.				
Education					
Junior high school or below	-537.354	-970.315	-104.392	5.917	0.015
High school / technical secondary school	-107.020	-463.535	249.495	0.346	0.556
College degree or above	Ref.				
Family monthly per capita income					
< 5000	-49.486	-466.970	367.998	0.054	0.816
5000–9999	-214.944	-606.449	176.561	1.158	0.282
≥10000	Ref.				
Cities					
First-tier	272.708	-161.197	706.613	1.517	0.218
New first-tier	56.263	-355.940	468.467	0.072	0.789

Second-tier	133.621	-267.717	534.960	0.426	0.514
Third- or fourth-tier	Ref.				
Pre-pregnancy BMI					
Underweight	533.043	-29.698	1095.784	3.447	0.063
Normal	182.978	-171.365	537.322	1.024	0.311
Overweight/obesity	Ref.				
Gravidity					
1	151.633	-235.526	538.793	0.589	0.443
≥2	Ref.				
Parity					
0	-365.748	-787.466	55.970	2.889	0.089
≥1	Ref.				

(q) Sodium(mg)

Variables	β	95% CI		Wald value	P
		Lower Limit	Upper Limit		
NVP group					
NVP	-235.671	-803.668	332.325	0.661	0.416
Non-NVP	Ref.				
Age (years)					
< 30	19.178	-455.834	494.191	0.006	0.937
≥30	Ref.				
Education					
Junior high school or below	410.750	-234.652	1056.152	1.556	0.212
High school / technical secondary school	-405.684	-937.130	125.761	2.238	0.135
College degree or above	Ref.				
Family monthly per capita income					
< 5000	-245.863	-868.193	376.467	0.600	0.439
5000–9999	-95.971	-679.575	487.633	0.104	0.747
≥10000	Ref.				
Cities					
First-tier	-549.040	-1195.848	97.767	2.768	0.096
New first-tier	-379.287	-993.745	235.172	1.464	0.226
Second-tier	-75.688	-673.950	522.574	0.061	0.804
Third- or fourth-tier	Ref.				
Pre-pregnancy BMI					
Underweight	88.323	-750.537	927.183	0.043	0.837
Normal	260.792	-267.416	789.001	0.936	0.333
Overweight/obesity	Ref.				
Gravidity					
1	375.562	-201.565	952.688	1.627	0.202
≥2	Ref.				
Parity					

0	-212.796	-841.437	415.846	0.440	0.507
≥1	Ref.				

(r) Magnesium(mg)

Variables	β	95% CI		Wald value	P
		Lower Limit	Upper Limit		
NVP group					
NVP	-40.536	-88.597	7.526	2.733	0.098
Non-NVP	Ref.				
Age (years)					
< 30	-20.816	-61.009	19.378	1.030	0.310
≥30	Ref.				
Education					
Junior high school or below	-61.144	-115.755	-6.533	4.816	0.028
High school / technical secondary school	-0.480	-45.449	44.488	0.000	0.983
College degree or above	Ref.				
Family monthly per capita income					
< 5000	-2.374	-55.033	50.284	0.008	0.930
5000–9999	-27.067	-76.448	22.315	1.154	0.283
≥10000	Ref.				
Cities					
First-tier	-10.384	-65.114	44.345	0.138	0.710
New first-tier	-37.976	-89.969	14.016	2.049	0.152
Second-tier	1.124	-49.498	51.746	0.002	0.965
Third- or fourth-tier	Ref.				
Pre-pregnancy BMI					
Underweight	55.590	-15.391	126.570	2.356	0.125
Normal	1.115	-43.579	45.810	0.002	0.961
Overweight/obesity	Ref.				
Gravidity					
1	6.232	-42.601	55.066	0.063	0.802
≥2	Ref.				
Parity					
0	-30.540	-83.732	22.653	1.266	0.260
≥1	Ref.				

(s) Iron(mg)

Variables	β	95% CI		Wald value	P
		Lower Limit	Upper Limit		
NVP group					
NVP	-6.729	-11.502	-1.956	7.634	0.006
Non-NVP	Ref.				
Age (years)					

< 30	-2.896	-6.888	1.096	2.022	0.155
≥30	Ref.				
Education					
Junior high school or below	-2.826	-8.250	2.598	1.043	0.307
High school / technical secondary school	0.288	-4.178	4.754	0.016	0.899
College degree or above	Ref.				
Family monthly per capita income					
< 5000	-1.125	-6.355	4.104	0.178	0.673
5000–9999	-3.649	-8.554	1.255	2.127	0.145
≥10000	Ref.				
Cities					
First-tier	-1.552	-6.987	3.883	0.313	0.576
New first-tier	0.411	-4.753	5.574	0.024	0.876
Second-tier	1.569	-3.458	6.597	0.374	0.541
Third- or fourth-tier	Ref.				
Pre-pregnancy BMI					
Underweight	4.112	-2.937	11.162	1.307	0.253
Normal	0.656	-3.783	5.094	0.084	0.772
Overweight/obesity	Ref.				
Gravidity					
1	-1.017	-5.867	3.833	0.169	0.681
≥2	Ref.				
Parity					
0	0.804	-4.478	6.087	0.089	0.765
≥1	Ref.				

(t) Zinc(mg)

Variables	β	95% CI		Wald value	P
		Lower Limit	Upper Limit		
NVP group					
NVP	-2.150	-3.465	-0.835	10.264	0.001
Non-NVP	Ref.				
Age (years)					
< 30	0.247	-0.853	1.347	0.193	0.660
≥30	Ref.				
Education					
Junior high school or below	-1.656	-3.150	-0.162	4.717	0.030
High school / technical secondary school	-0.531	-1.762	0.699	0.716	0.397
College degree or above	Ref.				
Family monthly per capita income					
< 5000	-1.149	-2.590	0.292	2.442	0.118
5000–9999	-1.026	-2.377	0.325	2.215	0.137
≥10000	Ref.				

Cities					
First-tier	-0.286	-1.783	1.212	0.140	0.708
New first-tier	-0.735	-2.158	0.688	1.025	0.311
Second-tier	0.621	-0.764	2.006	0.772	0.380
Third- or fourth-tier	Ref.				
Pre-pregnancy BMI					
Underweight	1.219	-0.723	3.161	1.513	0.219
Normal	-0.500	-1.723	0.723	0.643	0.423
Overweight/obesity	Ref.				
Gravidity					
1	0.074	-1.263	1.410	0.012	0.914
≥2	Ref.				
Parity					
0	-0.908	-2.364	0.547	1.496	0.221
≥1	Ref.				

(u) Selenium (mg)

Variables	β	95% CI		Wald value	P
		Lower Limit	Upper Limit		
NVP group					
NVP	-14.882	-28.265	-1.499	4.750	0.029
Non-NVP	Ref.				
Age (years)					
< 30	2.789	-8.403	13.981	0.239	0.625
≥30	Ref.				
Education					
Junior high school or below	-8.605	-23.812	6.601	1.230	0.267
High school / technical secondary school	-3.039	-15.561	9.482	0.226	0.634
College degree or above	Ref.				
Family monthly per capita income					
< 5000	-10.125	-24.788	4.538	1.832	0.176
5000–9999	-9.846	-23.596	3.905	1.970	0.160
≥10000	Ref.				
Cities					
First-tier	0.467	-14.773	15.706	0.004	0.952
New first-tier	0.925	-13.552	15.403	0.016	0.900
Second-tier	-4.175	-18.271	9.921	0.337	0.562
Third- or fourth-tier	Ref.				
Pre-pregnancy BMI					
Underweight	15.740	-4.024	35.505	2.436	0.119
Normal	1.279	-11.167	13.724	0.041	0.840
Overweight/obesity	Ref.				
Gravidity					

1	2.262	-11.336	15.860	0.106	0.744
≥2	Ref.				
Parity					
0	-15.523	-30.334	-0.711	4.219	0.040
≥1	Ref.				

(v) Copper (mg)

Variables	β	95% CI		Wald value	P
		Lower Limit	Upper Limit		
NVP group					
NVP	-0.295	-0.949	0.359	0.781	0.377
Non-NVP	Ref.				
Age (years)					
< 30	0.164	-0.383	0.711	0.346	0.556
≥30	Ref.				
Education					
Junior high school or below	-0.633	-1.377	0.110	2.789	0.095
High school / technical secondary school	0.221	-0.391	0.834	0.503	0.478
College degree or above	Ref.				
Family monthly per capita income					
< 5000	-0.072	-0.789	0.645	0.039	0.844
5000–9999	0.121	-0.551	0.793	0.125	0.724
≥10000	Ref.				
Cities					
First-tier	-0.292	-1.037	0.453	0.589	0.443
New first-tier	0.422	-0.286	1.129	1.363	0.243
Second-tier	0.110	-0.579	0.799	0.097	0.755
Third- or fourth-tier	Ref.				
Pre-pregnancy BMI					
Underweight	0.628	-0.338	1.594	1.622	0.203
Normal	0.298	-0.310	0.907	0.924	0.336
Overweight/obesity	Ref.				
Gravidity					
1	0.249	-0.415	0.914	0.540	0.462
≥2	Ref.				
Parity					
0	-0.837	-1.561	-0.113	5.139	0.023
≥1	Ref.				

(w) Manganese (mg)

Variables	β	95% CI		Wald value	P
		Lower Limit	Upper Limit		
NVP group					

NVP	-0.560	-2.373	1.252	0.367	0.545
Non-NVP	Ref.				
Age (years)					
< 30	-1.185	-2.701	0.331	2.346	0.126
≥30	Ref.				
Education					
Junior high school or below	-0.397	-2.457	1.663	0.143	0.706
High school / technical secondary school	0.154	-1.543	1.850	0.031	0.859
College degree or above	Ref.				
Family monthly per capita income					
< 5000	-0.636	-2.622	1.350	0.394	0.530
5000–9999	-0.740	-2.603	1.122	0.607	0.436
≥10000	Ref.				
Cities					
First-tier	0.904	-1.160	2.968	0.737	0.391
New first-tier	-0.138	-2.099	1.823	0.019	0.890
Second-tier	0.662	-1.247	2.572	0.462	0.497
Third- or fourth-tier	Ref.				
Pre-pregnancy BMI					
Underweight	0.828	-1.849	3.505	0.367	0.544
Normal	0.680	-1.006	2.366	0.625	0.429
Overweight/obesity	Ref.				
Gravidity					
1	-0.775	-2.617	1.067	0.680	0.410
≥2	Ref.				
Parity					
0	0.010	-1.996	2.016	0.000	0.992
≥1	Ref.				

Table S3 Comparison of dietary nutrient intake of pregnant women with different severity of nausea and vomiting

	Non-NVP	Mild NVP		Moderate NVP		Severe NVP		Spearman's P
	Median(P25,P75)	Median(P25,P75)	P^a	Median(P25,P75)	P^a	Median(P25,P75)	P^a	
Energy	1897.7(1449.5,2390.7)	1699.5(1354.7,2115.5)	0.069	**1539.9(1201.3,1932.2)**	**0.006**	**1230.5(774.5,1897.1)**	**0.001**	**＜0.001*****
Protein	63.4(42,86)	51.6(37.7,70.7)	0.094	**49.9(35.8,63.3)**	**0.040**	**42.4(22.7,66.9)**	**0.011**	**0.004****
Fat	77.9(58,102.5)	**55.9(42.1,75.9)**	＜0.001	**56.5(40.8,79.8)**	**0.004**	**49.9(29.9,70.7)**	**0.001**	**0.001****
Carbohydrate	220.6(167,279.9)	230(171.1,299)	0.875	185.1(132.8,269.8)	0.102	**147.8(91.5,273.5)**	**0.013**	**0.004****
Dietary fiber	8.6(6.1,14.7)	9.1(5.8,13.1)	0.726	10.5(5.2,14.3)	0.881	**6.5(3.9,10.3)**	**0.029**	0.174
Cholesterol	428(112.4,768)	349.8(73.8,583.2)	0.167	**301(94.4,452.3)**	**0.049**	309.4(67.5,562.6)	0.164	0.054
Vitamin A	399.7(175.5,603.9)	291.9(132.8,472.7)	0.061	273.5(150.8,427)	0.067	**222.4(74,350.2)**	**0.015**	**0.021***
Thiamin	0.8(0.6,1)	**0.7(0.4,1)**	**0.034**	**0.6(0.4,0.9)**	**0.018**	**0.5(0.4,0.7)**	＜0.001	**0.001****
Riboflavin	0.7(0.5,1.2)	**0.6(0.5,0.8)**	**0.020**	0.7(0.5,0.9)	0.103	**0.4(0.3,0.7)**	**0.001**	**0.004****
Niacin	10.9(6.4,16.3)	10.1(7,14.6)	0.671	9.1(6.6,12.9)	0.273	8.7(6.7,10.9)	0.063	**0.039***
Vitamin C	58(29.6,107.1)	56.9(27.5,98.5)	0.648	49.1(22.9,98.8)	0.568	36.6(16.1,96.7)	0.246	0.276
Vitamin E	31.1(20.9,47.8)	**22.4(15.1,34.8)**	**0.002**	24.1(17.1,37.7)	0.052	**19.2(14.5,31.7)**	**0.001**	**0.019***
Folate	224.8(138.8,347.1)	216.7(150.1,308.4)	0.592	193.6(135.4,300.9)	0.347	173.1(126.2,279.7)	0.145	0.134
Calcium	375.7(218.6,796.7)	324.3(213.5,515.8)	0.137	292.6(184,599.4)	0.183	**208.4(108.1,510.1)**	**0.018**	**0.022***
Phosphorus	816.2(667.3,1315.5)	**744.2(569.3,1043.1)**	**0.047**	747.7(541.8,999.1)	0.06	**534.7(370.1,849.7)**	**0.001**	**0.001****
Potassium	1816.2(1314.5,2975.2)	**1538.1(1125.3,2043.6)**	**0.048**	1719.9(1096.2,2309.6)	0.396	**1103.1(763.1,1737.8)**	**0.005**	**0.035***
Sodium	4132.5(3532.8,5237.1)	3995.3(3087.7,4965.6)	0.130	4045.8(3192.5,4769.1)	0.187	3700.3(2620.1,4834.2)	0.126	0.129
Magnesium	252.4(178.1,305.1)	227.1(174.9,309.1)	0.328	229.4(170.5,311.3)	0.586	182.1(121.4,284.9)	0.052	0.12
Iron	15.3(11.4,20.8)	13.6(9.7,18.8)	0.086	12.6(9.1,18.1)	0.05	**9.7(5.5,17.3)**	**0.001**	**0.001****
Zinc	8(6,12.9)	7(5.1,10.2)	0.093	**6.7(4.8,8.7)**	**0.021**	**5.2(3.5,8.6)**	**0.004**	**0.001****
Selenium	33.8(24.1,58.3)	32.1(20.8,44.1)	0.174	28.1(23,44.6)	0.078	**19.6(10.4,45)**	**0.016**	**0.011***
Copper	1.4(1,2.3)	1.3(0.9,1.8)	0.235	1.3(0.9,2.1)	0.524	1(0.6,1.9)	0.153	0.275
Manganese	3.1(2.3,3.9)	2.7(2,4.1)	0.524	2.7(1.8,4.4)	0.396	**2.3(1.6,3.4)**	**0.021**	**0.049***

[a] Compared to the non-NVP group.

* *P* < 0.05; ** *P* < 0.01; *** *P* < 0.001

NVP: Nausea and vomiting in pregnancy;

Table S4 Comparison of dietary nutrient intake of pregnant women with different severity of nausea and vomiting after adjusting for demographic characteristics

	Non-NVP	Mild NVP		Moderate NVP		Severe NVP	
		Adjusted β(95%CI)[a]	P	Adjusted β(95%CI)[a]	P	Adjusted β(95%CI)[a]	P
Energy(kcal)	Ref.	-216.3(-436.9,4.2)	0.055	**-336.9(-585,-88.9)****	**0.008**	**-585.8(-891.4,-280.3)*****	**＜0.001**
Protein(g)	Ref.	**-15.9(-26.9,-4.8)****	**0.005**	**-20.7(-33.1,-8.3)****	**0.001**	**-25.9(-41.2,-10.6)****	**0.001**
Fat(g)	Ref.	**-15(-24.7,-5.2)****	**0.003**	**-15.6(-26.6,-4.6)****	**0.005**	**-24.7(-38.2,-11.1)*****	**＜0.001**
Carbohydrate(g)	Ref.	-13.2(-46.7,20.3)	0.440	-34.4(-72.1,3.3)	0.073	**-61.8(-108.2,-15.3)****	**0.009**
Dietary fiber(g)	Ref.	-2.5(-5.4,0.3)	0.079	-2(-5.2,1.2)	0.218	-3.9(-7.8,0.1)	0.054
Cholesterol(mg)	Ref.	-59.4(-174.9,56.2)	0.314	**-131.7(-261.6,-1.7)***	**0.047**	-116.9(-277,43.2)	0.152
Vitamin A(μgREA)	Ref.	-66.8(-342,208.4)	0.634	-146.1(-455.6,163.3)	0.355	-111.6(-492.8,269.6)	0.566
Thiamin(mg)	Ref.	**-0.2(-0.3,0)***	**0.027**	**-0.2(-0.4,0)***	**0.013**	**-0.3(-0.5,-0.1)****	**0.004**
Riboflavin(mg)	Ref.	**-0.3(-0.5,-0.1)****	**0.001**	**-0.3(-0.5,-0.1)****	**0.005**	**-0.5(-0.7,-0.2)****	**0.001**
Niacin(mg)	Ref.	-1.5(-4.2,1.2)	0.281	-1.5(-4.6,1.5)	0.323	**-4.6(-8.4,-0.8)***	**0.018**
Vitamin C(mg)	Ref.	2.8(-36.1,41.6)	0.889	-2(-45.7,41.8)	0.93	27.1(-26.8,81)	0.324
Vitamin E(mg)	Ref.	**-7(-12.9,-1.1)***	**0.021**	-5.3(-12,1.3)	0.116	**-12.4(-20.6,-4.2)****	**0.003**
Folate(μgDFE)	Ref.	-24.9(-79.8,30.1)	0.375	-53.8(-115.9,8.3)	0.089	-54.1(-130.2,22)	0.163
Calcium(mg)	Ref.	**-213.7(-347.1,-80.3)****	**0.002**	**-179.2(-329.2,-29.1)***	**0.019**	**-190.8(-375.6,-6)***	**0.043**
Phosphorus(mg)	Ref.	**-233(-375.8,-90.2)****	**0.001**	**-241.7(-402.3,-81.1)****	**0.003**	**-395.4(-593.2,-197.6)*****	**＜0.001**
Potassium(mg)	Ref.	**-512(-912.6,-111.4)***	**0.012**	-264(-714.5,186.5)	0.251	**-676.2(-1231.1,-121.2)***	**0.017**
Sodium(mg)	Ref.	-197.5(-797.1,402.1)	0.519	-209.2(-883.5,465.1)	0.543	-463(-1293.5,367.6)	0.275
Magnesium(mg)	Ref.	-46.5(-97.1,4.1)	0.072	-19.2(-76.1,37.7)	0.509	-60.4(-130.5,9.7)	0.091
Iron(mg)	Ref.	**-6.2(-11.2,-1.1)***	**0.016**	**-6.4(-12,-0.7)***	**0.027**	**-9.9(-16.9,-3)****	**0.005**

Zinc(mg)	Ref.	**-1.8(-3.2,-0.4)***	**0.011**	**-2.4(-3.9,-0.8)****	**0.003**	**-3.3(-5.2,-1.4)****	**0.001**
Selenium(mg)	Ref.	-12.5(-26.6,1.6)	0.083	**-16.6(-32.5,-0.7)***	**0.040**	**-21.8(-41.4,-2.3)***	**0.028**
Copper(mg)	Ref.	-0.2(-0.8,0.5)	0.660	-0.4(-1.2,0.4)	0.323	-0.7(-1.7,0.2)	0.145
Manganese(mg)	Ref.	-1.1(-3,0.8)	0.265	1(-1.1,3.1)	0.358	-1.7(-4.3,1)	0.213

[a] Adjustment factors include age, education, income, city, pre-pregnancy BMI, gravidity and parity.

* $P < 0.05$; ** $P < 0.01$; *** $P < 0.001$

NVP: Nausea and vomiting in pregnancy;

Table S5 Comparison of average daily food intake of pregnant women with different severities of nausea and vomiting.

	Non-NVP	Mild NVP		Moderate NVP		Severe NVP		Spearman's P
	Median(P25,P75)	Median(P25,P75)	P[a]	Median(P25,P75)	P[a]	Median(P25,P75)	P[a]	
Cereals	198.0(120.4,289)	238(122.5,349.7)	0.23	182.1(102.3,272.9)	0.416	176.7(111.3,295.1)	0.715	0.181
Potatoes	32.7(14.0,70..0)	23.3(7.9,52.5)	0.103	23.3(6.7,55.0)	0.145	14.0(0.0,89.3)	0.218	0.146
Fresh vegetables	160(58.3,300.0)	140(60,250)	0.408	150.0(54.2,232.5)	0.426	**75.0(21.7,200.0)**	**0.030**	0.053
Pickled and fermented vegetables	0.3(0.0,4.7)	0.1(0.0,4.7)	0.467	0.2(0.0,3.3)	0.348	0.1(0.0,2.3)	0.346	0.300
Mushroom and algae	11.7(4.1,20.1)	7.2(1.7,19.0)	0.072	**5.7(0.9,13.7)**	**0.019**	6.6(2.4,28.9)	0.355	0.110
Soybeans and their products	20.0(8.2,48.0)	18.9(6.7,46.2)	0.658	12.5(3.9,35.0)	0.12	20.6(11.3,35.7)	0.773	0.213
Nuts and seeds	22.7(7.9,40.0)	**11.7(1.3,26.5)**	**0.005**	**9.3(1.1,21.9)**	**＜0.001**	14.5(1.6,25.0)	0.054	**0.004****
Fruits	300.0(200.0,500.0)	250.0(152.5,400)	0.189	260(150.0,400.0)	0.188	300.0(180.0,500.0)	0.797	0.551
Poultry meat	38.5(14.2,97.5)	**30.0(4.7,50.0)**	**0.049**	**11.7(0.0,37.5)**	**＜0.001**	**6.4(0.0,52.5)**	**0.002**	**＜0.001*****
Livestock meat	11.0(3.3,43.8)	11.7(0.0,28.9)	0.314	**5.3(0.0,23.3)**	**0.028**	**1.7(0.0,16.6)**	**0.004**	**0.002****
Aquatic products	14.6(3.8,68.8)	16.3(3.3,35.0)	0.434	10.8(1.0,23.3)	0.077	8.2(0.0,50.6)	0.242	**0.039***
Eggs	60.0(46.4,60.0)	**50.0(21.0,60.0)**	**0.013**	**45.3(18.4,60.0)**	**0.022**	**49.0(9.4,60.0)**	**0.037**	**0.026***
Dairy products	250.0(101.3,295.4)	200.0(61.7,291.7)	0.267	**91.3(11.7,250.0)**	**0.006**	**46.7(0,0,225.0)**	**0.002**	**＜0.001*****
Soups	44.2(0.0,186.7)	43.3(0.0,116.7)	0.427	23.3(0.0,93.3)	0.124	93.3(5.2,140)	0.811	0.506
Snacks and beverages	5.0(0.0,46.8)	1.4(0.0,24.3)	0.217	1.2(0.0,16.7)	0.134	5.8(0.0,32.4)	0.880	0.586

[a] Compared to the non-NVP group.

* P < 0.05; ** P < 0.01; *** P < 0.001

NVP: Nausea and vomiting in pregnancy;

Table S6 Comparison of d average daily food intake of pregnant women with different severity of nausea and vomiting after adjusting for demographic characteristics

	Non-NVP	Mild NVP		Moderate NVP		Severe NVP	
		Adjusted β(95%CI)[a]	P	Adjusted β(95%CI)[a]	P	Adjusted β(95%CI)[a]	P
Cereals	Ref.	49.8(-12.5,112.1)	0.117	-21.2(-90.7,48.2)	0.549	-8.5(-93.9,76.9)	0.845
Potatoes	Ref.	-5.6(-22.2,10.9)	0.506	-4.9(-23.4,13.6)	0.605	3.6(-19.4,26.6)	0.76
Fresh vegetables	Ref.	-20.9(-98.3,56.6)	0.597	-38.9(-125.5,47.6)	0.378	-41.8(-148.4,64.7)	0.442
Pickled and fermented vegetables	Ref.	-2.4(-6.5,1.7)	0.254	-3.8(-8.4,0.8)	0.104	-4.4(-10,1.3)	0.131
Mushroom and algae	Ref.	-2.9(-10,4.2)	0.421	-4.3(-12.2,3.6)	0.284	0.3(-9.4,9.9)	0.955
Soybeans and their products	Ref.	-7.2(-21.2,6.7)	0.309	**-19.1(-34.9,-3.4)***	**0.017**	-10.2(-29.5,9.1)	0.299
Nuts and seeds	Ref.	**-8.9(-17.3,-0.4)***	**0.039**	**-17.2(-26.7,-7.7)*****	**＜0.001**	**-12.3(-23.9,-0.6)***	**0.039**
Fruits	Ref.	-26.5(-117.2,64.3)	0.568	-35.2(-137,66.6)	0.498	-39(-163.9,85.9)	0.54
Poultry meat	Ref.	-10.2(-31.5,11.1)	0.349	**-28.3(-52.2,-4.4)***	**0.020**	**-30.7(-60.1,-1.2)***	**0.041**
Livestock meat	Ref.	-3.7(-15.1,7.8)	0.529	-12.2(-25,0.6)	0.062	**-18.4(-34.2,-2.6)***	**0.022**
Aquatic products	Ref.	-3.7(-18.1,10.7)	0.613	**-18(-34.2,-1.9)***	**0.029**	-10.5(-30.4,9.4)	0.299
Eggs	Ref.	-8.6(-19.1,2)	0.112	-9.5(-21.4,2.4)	0.119	-11.1(-25.7,3.5)	0.137
Dairy products	Ref.	-18.8(-71.2,33.6)	0.482	**-87.1(-145.6,-28.6)****	**0.004**	**-85.7(-158.1,-13.4)***	**0.020**
Soups	Ref.	-34.1(-71.6,3.5)	0.076	**-46.1(-88.1,-4)***	**0.032**	-13.8(-66.2,38.7)	0.607
Snacks and beverages	Ref.	10.4(-28.1,49)	0.596	3.3(-39.9,46.5)	0.881	-7.5(-60.3,45.4)	0.782

[a] Adjustment factors include age, education, income, city, pre-pregnancy BMI, gravidity and parity.

* P < 0.05; ** P < 0.01; *** P < 0.001

NVP: Nausea and vomiting in pregnancy;
